# Thioredoxin Delays Photoreceptor Degeneration, Oxidative and Inflammation Alterations in Retinitis Pigmentosa

**DOI:** 10.3389/fphar.2020.590572

**Published:** 2020-12-23

**Authors:** Roberto Gimeno-Hernández, Antolin Cantó, Angel Fernández-Carbonell, Teresa Olivar, Vicente Hernández-Rabaza, Inmaculada Almansa, María Miranda

**Affiliations:** Departamento Ciencias Biomédicas, Universidad Cardenal Herrera-CEU, CEU Universities, Valencia, Spain

**Keywords:** retina, thioredoxin, glutathione, vascular endothelial growth factor, glia, hepatic growth factor

## Abstract

Retinitis pigmentosa (RP) is an inherited ocular disorder with no effective treatment. RP onset and progression trigger a cascade of retinal disorders that lead to the death of photoreceptors. After photoreceptors death, neuronal, glial and vascular remodeling can be observed in the retina. The purpose of this study was to study if thioredoxin (TRX) administration is able to decrease photoreceptor death in an animal model of RP (rd1 mouse), but also if it is able to modulate the retinal oxidative stress, glial and vascular changes that can be observed as the disease progresses. Wild type and rd1 mice received several doses of TRX. After treatment, animals were euthanized at postnatals days 11, 17, or 28. Glutathione (GSH) and other thiol compounds were determined by high performance liquid chromatography (HPLC). Glial fibrilary acidic protein (GFAP) and anti-ionized calcium binding adaptor molecule 1 (Iba1) were studied by immunohistochemistry. Vascular endothelial growth factor (VEGF) and hepatic growth factor (HGF) expression were determined by western blot. TRX administration significantly diminished cell death in rd1 mouse retinas and increased GSH retinal concentrations at postnatal day 11 (PN11). TRX was also able to reverse glial alterations at PN11 and PN17. No alterations were observed in retinal VEGF and HGF expression in rd1 mice. In conclusion, TRX treatment decreases photoreceptor death in the first stages of RP and this protective effect may be due in part to the GSH system activation and to a partially decrease in inflammation.

## Introduction

The name retinitis pigmentosa (RP) describes a heterogeneous group of degenerative and inherited ocular disorders that causes severe visual impairment (Wright et al., 2010; Daiger et al., 2013) and in many cases leads to total blindness. It is considered a rare disease with a worldwide prevalence of 1 in 4,000 individuals (Boughman et al., 1980). The most important RP clinical manifestations are nictalopia (also called night blindness), visual field loss, decrease in the ability to distinguish objects at low contrast (Lindberg et al., 1981) and photophobia.

RP onset and progression triggers a cascade of retinal disorders that lead to the death of photoreceptors. After photoreceptors death, a reorganization of the retinal circuits occurs, causing changes in morphology and in the establishment of new synapses between retinal cells. Although the mechanisms of photoreceptors death are not completely clear, three phases can be distinguished in the RP degeneration process (Jones and Marc, 2005): i) phase I: photoreceptors suffer a period of stress, shortening of rods and a disorganization of their synaptic contacts is observed; ii) phase II: death of photoreceptors takes place, Müller cells form glial fibrotic walls throughout the retina and death of other retinal neurons can also occur; iii) phase III: neuronal, glial and vascular remodeling of the retina occurs (Jones et al., 2016). In this last stage of the disease, neural cells dye progressively, and Müller cells fill in the spaces left by them. This process is accompanied by a decrease in retinal blood flow in response to a reduced metabolic demand (Grunwald et al., 1996). The disappearance of retinal blood vessels has been related with a decrease in vascular endothelial growth factor (VEGF) in aqueous humor of patients with RP (Salom et al., 2008). VEGF was initially identified as a vascular permeability factor and recently it has been shown to influence the growth and survival of neurons (Sondell et al., 1999; Jin et al., 2000). Hepatic growth factor (HGF) plays also a role in ocular angiogenesis and neuroprotection of retinal neurons (Bussolino et al., 1992; Salom et al., 2010; Karamali et al., 2019; Lorenc et al., 2020), however it is still unknown whether alterations of this growth factor may be relevant in RP or not.

The retina is extremely rich in membranes with polyunsaturated lipids (Panfoli et al., 2012). This feature makes it especially sensitive to oxidative stress because they may suffer peroxidation. In addition, the rods are very active cells with high oxygen consumption. During the progression of the RP, when the rods die, oxygen consumption in the retina decreases and oxygen retinal concentration increases. Therefore, a situation of hyperoxia occurs that could induce oxidative damage. In this sense, antioxidant therapy has been suggested as a possible way to slow down the degenerative process in RP because they may decrease oxidative stress. On the other hand, antioxidants have some important advantages as their safety when being used in adult humans, being able to improve the vision, at least transiently. Regarding the use of important antioxidants: curcumin (Vasireddy et al., 2011), tauroursodeoxycholic acid (TUDCA) (Fernández-Sánchez et al., 2011), n-acetylcysteine (Lee et al., 2011; Yoshida et al., 2013), lutein (Berson et al., 2010), chlorogenic acid (Shin and Yu, 2014), or combinations of antioxidants such as lutein, zeaxanthin, alpha lipoic acid and glutathione (Sanz et al., 2007) have been studied in humans and animal models as possible therapies for RP.

Thioredoxin (TRX) family proteins contain the active center Cysteine-Glycine-Proline-Cysteine. They also present oxidized cysteine groups (Powis and Montfort, 2001). The TRX system participates in a wide range of functions within the cell, including protection against oxidative stress (Holmgren, 2000), DNA precursor synthesis, proliferation, regulation and cell differentiation, cell death control and immune system modulation (Gromer et al., 2004; Arnér and Holmgren, 2006). It also has anti-inflammatory functions (Nakamura et al., 2001). Thioredoxin is antioxidant because it facilitates the reduction of other proteins by cysteine thiol-disulfide exchange.

Levéillard et al. identified the rod-derived cone viability factor (RdCVF)) in 2004 (Léveillard et al., 2004). This factor, released by rods, is key in the conservation of cones viability and may protect them from death (Scholl et al., 2016). RdCVF is a truncated TRX type protein specifically expressed by photoreceptors (Léveillard and Sahel, 2010). The nucleoredoxin-like gene also known as NXNL1 encodes RdCVF. NXNL1 encodes for two proteins, one is RdCVF that does not have TRX (Chalmel et al., 2007). In addition, alternative processing of the gene results in a protein with TRX activity known as RdCVFL (thioredoxin-like protein rod-derived cone viability factor).

However, it has not been studied if TRX external administration may have a protective effect in RP. The main purpose of this study was to administrate TRX in an experimental RP animal model. It is well known that TRX is able to pass brain blood barrier and therefore it may pass the retinal blood barrier, and though we have not determined TRX retinal concentration we have determined its direct effect (such as the decrease in photoreceptor death and the increase in glutathione). We studied if TRX was able to decrease photoreceptor death and to modulate the retinal oxidative stress (determining TRX effect on the retinal concentration of one of the major intracellular antioxidants, glutathione), and glial and vascular changes that can be observed as the disease progress. Our study was performed at three different postnatal days: 11, 17, and 28.

The animal model used in this study was the rd1 mouse, which was first described by Keeler in 1924 (Keeler, 1924) and shows a rapid degeneration of photoreceptors. The disorder in this animal model is caused by a mutation in the gene that encodes the beta subunit of phosphodiesterase-6 (PDE6) of the rods (Lolley et al., 1980; Bowes et al., 1990). This enzymatic alteration causes the accumulation of cGMP. This accumulation makes the calcium channels generate an abnormal inflow (Gregory and Bird, 1995; Fox et al., 2003) that triggers a cascade of events that finally leads to the death of photoreceptor cells (Adler, 1996; Travis, 1998). The degeneration of photoreceptors, specifically of the rods, in rd1 mice is maximum between postnatal days 11 and 13 (PN11-13) (Paquet-Durand et al., 2006). At PN17, only a small percentage of the rods remain while 75% of the cones persist (Carter-Dawson et al., 1978). At PN28, the degeneration of the rods is complete, and the vascular system is affected (Blanks and Johnson, 1986).

## Materials and Methods

Control and rd1 mice were used in this study. Mice were housed in the facilities of the Research Unit of CEU Cardenal Herrera University. The animals were kept in cages under controlled conditions of temperature (20°C) and humidity (60%) and were housed under standard (12 h) cyclic lighting and had free access to water and to a standard diet (Harlan Ibérica S.L. (Barcelona, Spain)). Handling and care of the animals were approved by the CEU Cardenal Herrera Universities Committee for Animal Experiments (reference 11/013) and were also performed in accordance with ARVO (Association for Research in Vision and Ophthalmology) Statement for the use of animals in Ophthalmic and Vision Research.

Day of birth was considered as post-natal day 0 (PN0). TRX treatment started at PN8 (5 mg/kg weight dissolved in 1 ml of saline). TRX (T0910) was purchased from Sigma-Aldrich (Madrid, Spain) and administered intraperitoneally. TRX dose and administration method were selected according to previous studies reporting its ability to cross blood brain barrier (BBB) (Tian et al., 2014; Wang et al., 2015). Moreover, TRX are expressed in various areas of the mouse, rat, and human brain as well as in the retina (Hanschmann et al., 2013).

**FIGURE 1:**
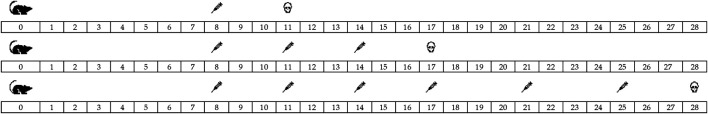
Experimental design for thioredoxin treatment of control and rd1 mice (TRX dose: 5 mg/kg weight; via: i.p.). Numbers indicate postnatal day. Group 1: control mice that received vehicle treatment at P8 and were euthanized at PN11; Group 2: control mice that received TRX treatment at P8 and were euthanized at PN11; Group 3: rd1 mice that received vehicle treatment at P8 and were euthanized at PN11; Group 4: rd1 mice that received TRX treatment at P8 and were euthanized at PN11; Group 5: control mice treated with vehicle at PN8, PN11, and PN14 and euthanized at PN17; Group 6: control mice treated with TRX at PN8, PN11, and PN14 and euthanized at PN17; Group 7: rd1 mice treated with vehicle at PN8, PN11, and PN14 and euthanized at PN17; Group 8: rd1 mice treated with TRX at PN8, PN11, and PN14 and euthanized at PN17; Group 9: control mice that received vehicle treatment at PN8, PN11, PN14, PN17, PN21, PN25 and were euthanized at PN28; Group 10: control mice that received TRX treatment at PN8, PN11, PN14, PN17, PN21, PN25 and were euthanized at PN28; Group 11: rd1 mice that received vehicle treatment at PN8, PN11, PN14, PN17, PN21, PN25 and were euthanized at PN28; Group 12: rd1 mice that received TRX treatment at PN8, PN11, PN14, PN17, PN21, PN25 and were euthanized at PN28.

Twelve experimental groups were used in this study (four groups for each postnatal day studied). Groups 1 to 4 were control and rd1 mice that received vehicle or TRX treatment at P8 and were euthanized at PN11. Groups 5 to 8 consisted on control and rd1 mice treated with vehicle or TRX at PN8, PN11 and PN14 and euthanized at PN17. Groups 9 to 12 control and rd1 mice that received vehicle or TRX treatment at PN8, PN11, PN14, PN17, PN21, PN25 and were euthanized at PN28 (Figure 1). The number of animals in each experimental group was at least 8.

### Histological and Immunofluorescence Studies

Firstly, eyes were enucleated and fixed by immersion in 4% paraformaldehyde (PFA) for 2 h. Secondly, eyes were washed with 0.1 M phosphate buffered saline pH 7.2 (PBS). Finally, they were cryoprotected in increasing concentrations of PBS-sucrose (10–20–30%) at 4°C. After embedding in Tissue Tek (Sakura Europe, Spain), the eyes were sectioned in a Leica CM 1850 UV Ag protect cryostat, (Leica Microsistemas SLU, Barcelona, Spain) (8 µm sections) on superfrost slides (Thermo Fisher Scientific, Braunschweig, Germany) and kept at −20°C. Only one retina per mice was used in histological and immunofluorescence studies.

#### Terminal Deoxinucleotidyl Transferase Assay

Detection of dying cells with the TUNEL assay was performed with an *in situ* detection kit (Roche Diagnostics, Mannheim, Germany) as reported previously (Benlloch-Navarro et al., 2019). Retinal images were taken with the Nikon DS-Fi1 camera attached to a Leica DM 2000 microscope. The Leica application Suite version 2.7.0 R1 (Leica Microsystems SLU, Barcelona, Spain) program was used.

#### Retinal Immunohistochemistry

Retinal tissue cryosections were rehydrated in PBS and merged with blocking solution: 5% of normal goat serum in PBS-BSA 1% and Triton 0.3%. Sections were incubated at 4°C over night in primary antibodies: anti-glial fibrillary acidic protein (anti-GFAP) (1:500, Dako cytomation, Denmark) and anti-ionized calcium binding adaptor molecule 1 (anti-Iba1 (1:2,000, Abcam, Cambridge, United Kingdom). Primary antibodies were detected with the fluorescence-conjugated secondary antibody Alexa Fluor 488 (Invitrogen, Life Technologies, Madrid, Spain). Sections were mounted with Vectashield mounting medium with DAPI (Vector, Burlingame, United States). Retinal images were viewed with a Nikon DS-Fi1 camera attached to a Leica DM 2000 microscope. Representative images were taken of three areas of the retina (far periphery, medium and central retina). To evaluate changes in macrogliosis, the percentage of area occupied by GFAP antibody labeling was measured in total retina. To evaluate microglial activation, total Iba-1 positive cells were counted per ONL area. All these measurements were made with the help of the program image processing Image J 1.45s.

### Biochemical Studies

#### Glutathione Assay

Both retinas per mice were homogenated together as described in Sanchez-Vallejo et al. (Sánchez-Vallejo et al., 2015). Reduced and oxidized glutathione (GSH, GSSG) as well as glutamate concentrations were quantified by means of the Reed method (Reed et al., 1980). Protein concentration was determined with the Lowry method (Lowry et al., 1951).

#### Western Blot

Two retinas of each animal were dissected and homogenized mechanically with 50 μL radioimmunoprecipitation (RIPA) buffer. Samples were then centrifuged at 13,000 rpm during 10 min at 4°C. Supernatant was removed and used for protein determination (Bradford, 1976). We used 75 μg of protein that were resolved during 1 h on 10–15% acrylamide:bisacrylamide gels at 200 V. The proteins were moved to nitrocellulose membranes (AmershamTM Hybond ECL (GE Healthcare Life Sciences, Barcelona, España) and blocked for 1 h with 0.01 M PBS-Tween 20 0.1% with 5% w/v non-fat milk. Membranes were probed with vascular endothelial growth factor (VEGF) and hepatic growth factor (HGF) antibodies (Santa Cruz Biotechnology, Santa Cruz, United States). The membrane was incubated over night at RT, and bound antibody was detected with a horseradish peroxidase coupled secondary anti-rabbit antibody (F (ab′) 2–HRP, goat anti-rabbit) (Santa Cruz Biotechnology, Santa Cruz, United States). The signal was recognized with the enhanced chemiluminescence (ECL) developing kit (Amersham Biosciences, Buckinghamshire, United Kingdom) and quantified by densitometry (Image Quant™TL, GE Healthcare Life Sciences, Barcelona, Spain).

### Statistical Analysis

The results are presented as mean values ± standard deviation. The analysis of variance (ANOVA) was used. When the ANOVA indicated a significant difference, the Bonferroni test was performed. Graphpad Prism 8 software package was used. The level of significance was stablished at *p* < 0.05.

## Results

### Thioredoxin Decreases Photoreceptor Death

**FIGURE 2:**
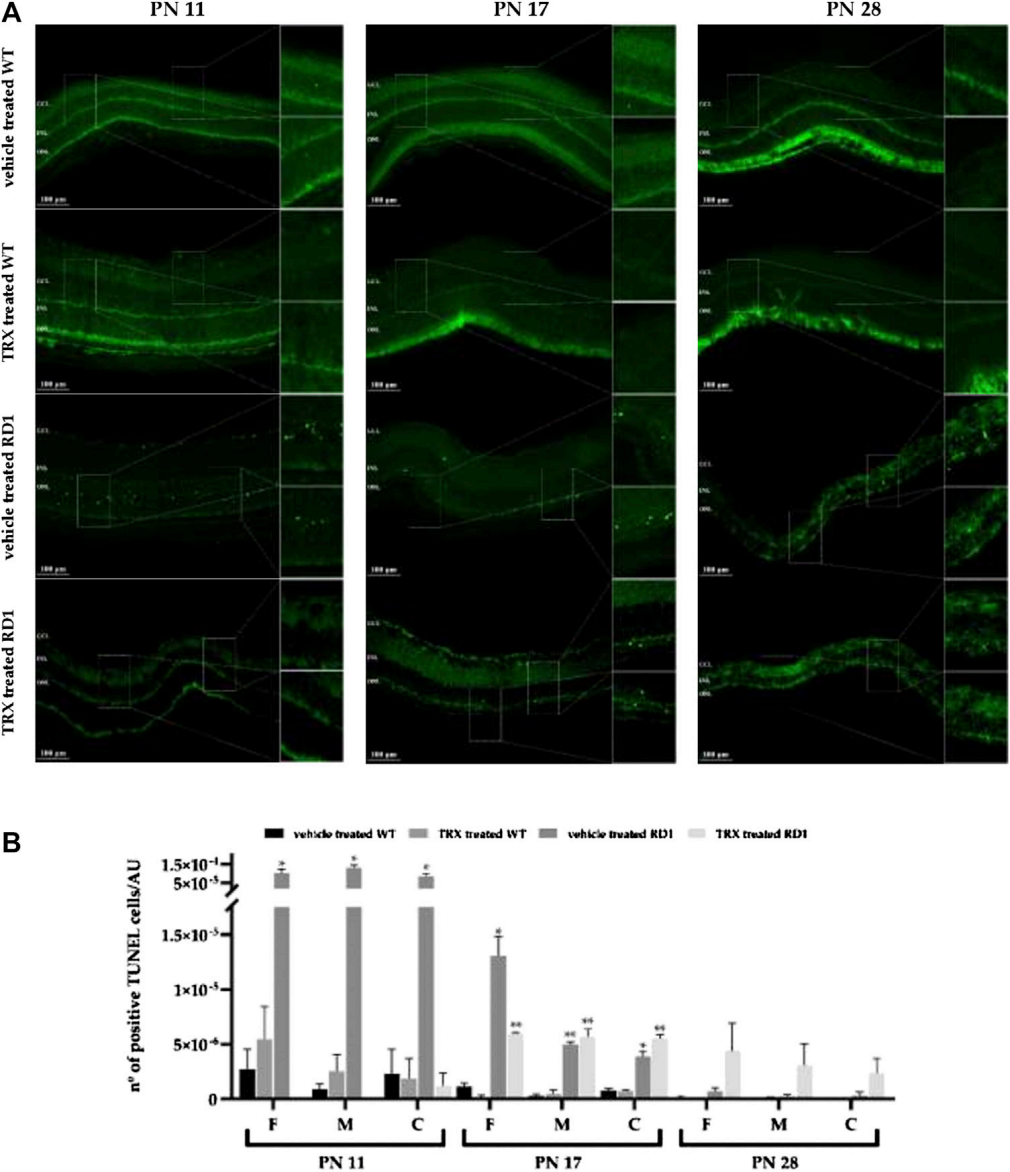
Detection of dying cells with TUNEL staining. **(A)** Central retinal TUNEL images from non-treated and treated with TRX wild type (WT) and rd1 mice at PN11, PN17 and PN28. **(B)** Quantification of TUNEL positive cells/arbitrary units of area (AUA) at PN11, PN17 and PN28 in three retinal regions (far periphery, medium and central retina). Mean values and standard errors are shown in the graph. The images are representative for observations for at least three different animals for each group (*p < 0.05 vs. WT vehicle treated, WT TRX treated and rd1 TRX treated; **p < 0.05 vs. WT vehicle treated and WT TRX treated). N = at least six in each experimental group. The TUNEL positive cells were counted manually in the outer nuclear layer (ONL) of three different parts of the retina: far periphery, medium and central retina. TUNEL positive cells from referred to the area of the ONL which was used.

Photoreceptor death and the possible neuroprotective effect of TRX was investigated with the TUNEL assay. At PN11 and PN17 a significant increase in the number of dead cells was found in the outer nuclear layer (ONL) of vehicle treated rd1 mice, when compared to both groups of wild type (WT) mice (vehicle and TRX treated) (Figures 2A,B). Moreover, TRX administration significantly diminish cell death in rd1 mouse retinas at PN11 in the three retinal areas studied (*p < 0.05). This result could show a neuroprotective effect of TRX treatment at this age (Figure 2B). However, at PN17 TRX was only able to decrease the number of TUNEL positive cells in the rd1 far periphery retina (*p < 0.05). In this sense, TRX treatment did not show any effect in photoreceptor death in the medium and central retina (Figure 2B). At PN28 no differences were observed between the number of TUNEL positive cells in wild type and rd1 mice. This result may be explained because at PN28 the degeneration in the rd1 retina is so advanced that there is almost no ONL left, and therefore, there are no more rods remaining and no death could be observed. No protective effect of TRX could be observed at PN28 (Figure 2B).

### Glutathione Metabolism Alterations in rd1 Retina: Thioredoxin Effect

**FIGURE 3:**
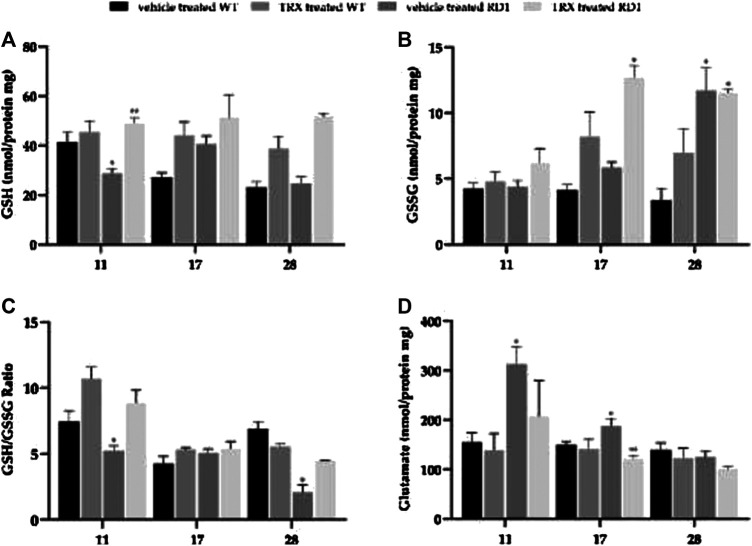
Retinal concentration of GSH, GSSG, GSH/GSG ratio and glutamate concentrations at PN11, PN17 and PN28. (*p < 0.05 vs. all the other groups; **p < 0.05 vs. WT vehicle treated). N = at least six in each experimental group.

Figure 3 shows the representation of the retinal concentration of GSH, GSSG and GSH/GSG ratio as well as glutamate concentrations in the four studied groups per each of the four ages studied (vehicle treated WT, TRX treated WT, vehicle treated rd1, TRX treated rd1 mice).

At PN11 there were no significant changes in retinal GSH concentrations when WT mice were treated with TRX, as observed in Figure 3A. GSH concentration was significantly decreased in rd1 retinas when compared with all the other groups (*p < 0.05), with values 28.74 ± 2.05 8 nmol/mg protein (Figure 3A). There is also a statistically significant increase (approximately 40% of increase) in the concentration of GSH in the group of rd1 mice treated with TRX compared to the rd1 group treated with the vehicle. When the retinal degeneration has progressed and the decrease in the ONL layer is more evident (PN17 and PN28) no differences could be observed between retinal GSH concentrations in WT and rd1 mice. However, repeated doses of TRX induced a significant increase in GSH concentrations in both groups of animals.

Figure 3B shows GSSG concentrations in the retina of the different animal groups at the three ages studied. At PN11 and PN17 no differences were observed between GSSG concentrations in WT and rd1 treated with vehicle. Again, the administration of TRX increased significantly this retinal parameter in rd1 treated animals. At PN28, an increase in GSSG concentration was observed in rd1 treated with vehicle or TRX animals when compared to WT treated with vehicle or TRX mice.

Probably, a better marker of the retinal antioxidant defenses is the GSH/GSSG ratio. In our study, the results obtained regarding this marker are shown in Figure 3C. Our results suggest that there is a significant alteration of GSH/GSSG ratio in rd1 mice at PN11 and PN28, and that this alteration is completely reverted with the administration of TRX.

Glutamate is the largest excitatory retinal neurotransmitter, but an excess of this transmitter may be toxic and may lead to degeneration. Our results show (Figure 3D) an increase in glutamate retinal concentrations in rd1 mice in comparison with WT animals but also in comparison with rd1 mice that where treated with TRX (*p < 0.05) at PN11 and PN17. No significant differences were observed at PN28, although the minimum concentration corresponds to rd1 group treated with TRX (100.95 ± 4.97 nmol/mg protein).

### Thioredoxin Treatment Partially Reduces Retinal Macro and Microgliosis in rd1 Mice

Similarly, to other retinal pathologies, inflammation plays an important role in RP. In this sense it is well known that the expression of glial fibrillary acidic protein (GFAP) is expressed at a low level in Müller glial cells in control animals. However, when the retina is damaged GFAP is strongly upregulated. Therefore, GFAP is a very sensitive marker for retinal inflammation (macrogliosis) and neurodegeneration.

**FIGURE 4:**
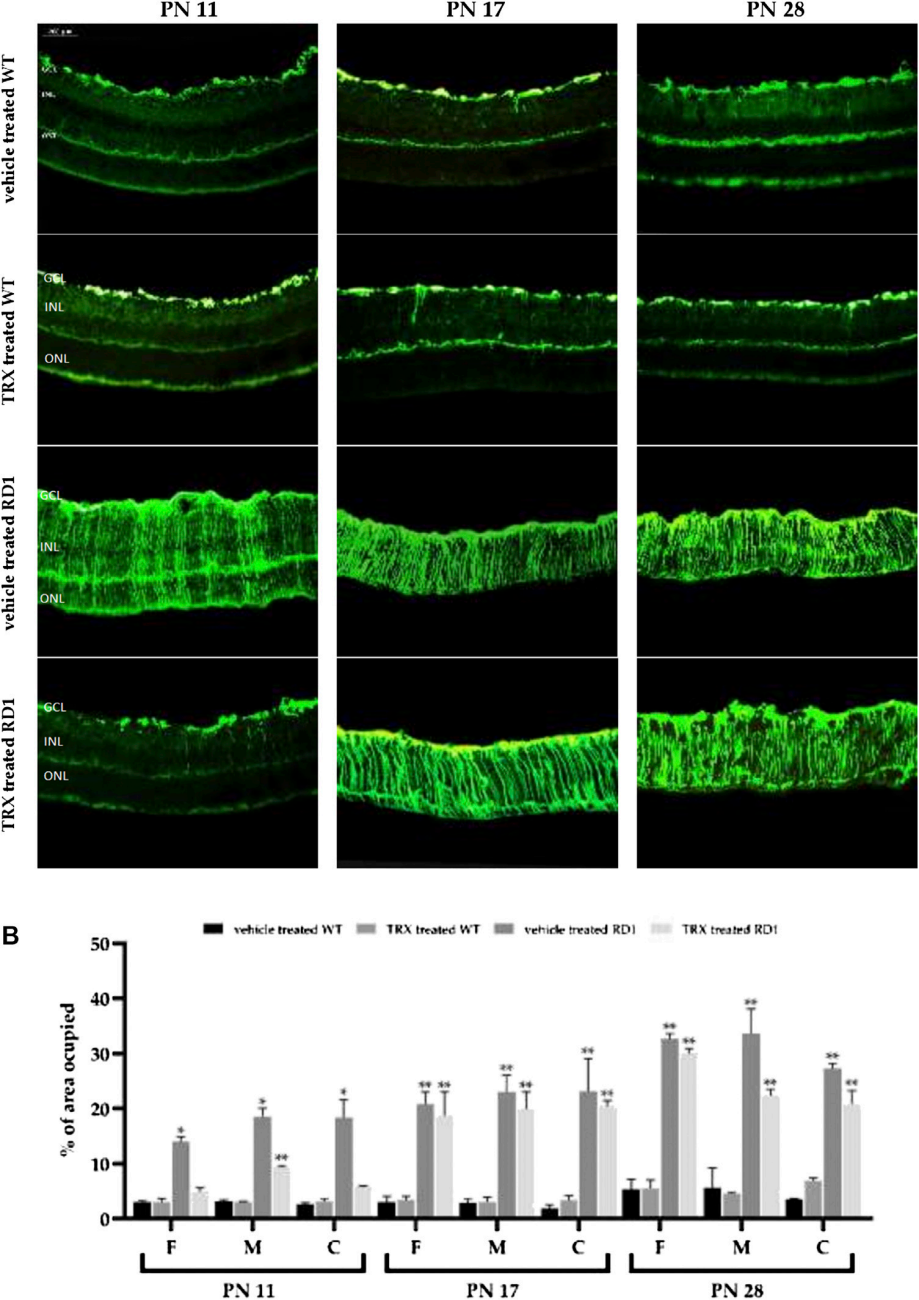
Retinal GFAP immunoreactivity. GFAP is increased in rd1 retina and partially restored after TRX administration. **(A)** Images of GFAP fluorescent labeling. **(B)** Graphic representation of the quantification of the area occupied by retinal GFAP staining. The bars represent the percentage of are occupied by the positive GFAP cells (n = 4) and the error bars represent the standard error of the mean (*p < 0.05 vs. WT vehicle treated, WT TRX treated and rd1 TRX treated; **p < 0.05 vs. WT vehicle treated and WT TRX treated). N = at least six in each experimental group.

In WT animals (treated with vehicle or TRX), retinal GFAP immunoreactivity was observed only next to the ganglion cell layer (GCL) (Figure 4A). In rd1 animals treated with vehicle, GFAP staining was found throughout the retina (Figure 4A). Quantification of the occupied area of the retina by the GFAP staining at PN11, PN17 and PN28 for the different experimental animal groups in each of the areas of the retina is shown in Figure 4B. We found statistically significant increases in these GFAP values in all areas of the retina at all the ages studied in rd1 treated with vehicle mice (*p < 0.05 vs. all other groups). Interestingly, we also observed that TRX treatment decreased GFAP reactivity in rd1 mice, but this effect was only observed at P11. No TRX effect was observed on the characteristic rd1 retinal macrogliosis at PN17 or PN28.

**FIGURE 5:**
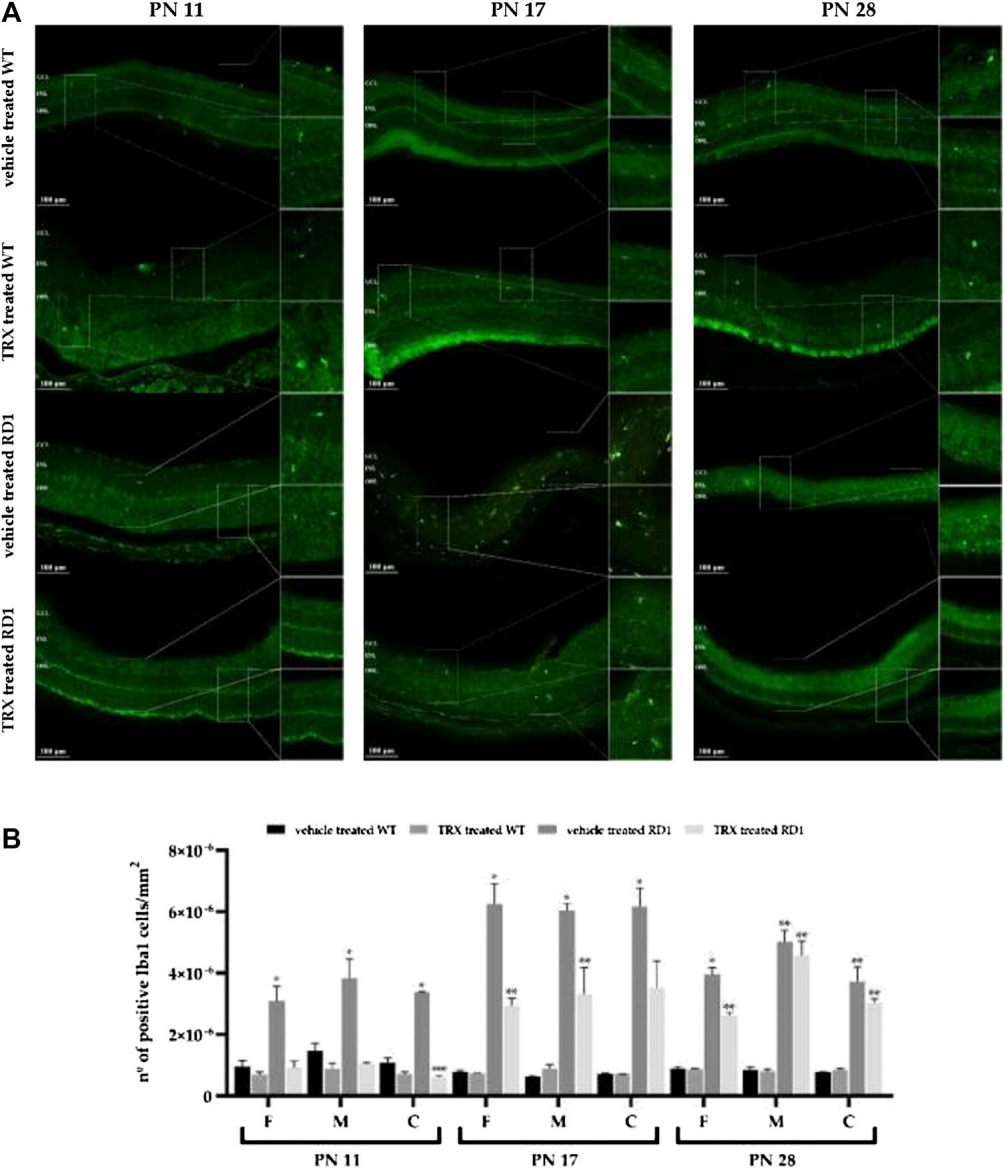
TRX effect on Iba1 expression. **(A)** Images of Iba1 fluorescent labeling. **(B)** Graphic representation of the quantification of number of Iba1 positive cells. The bars represent the number of Iba1 positive cells per units of area (n = 4) and the error bars represent the standard error of the mean (*p < 0.05 vs. WT vehicle treated, WT TRX treated and rd1 TRX treated; **p < 0.05 vs. WT vehicle treated and WT TRX treated; ***p < 0.05 vs. rd1 vehicle treated). N = at least six in each experimental group. The number of Iba1 cells were counted manually in the outer nuclear layer (ONL) of three different parts of the retina: far periphery, medium and central retina. TUNEL positive cells from referred to the area of the ONL which was used.

We have also studied whether TRX treatment was able to reduce microglial activation in rd1 retinas and, therefore, used the Iba1 antibody to identify microglial cells. Iba1 immunolabeling was upregulated in rd1 retinas compared to control retinas in all the areas and at all the ages studied (Figures 5A,B). These results are similar to those found for GFAP. TRX significantly reduced Iba1-positive immunolabelling in rd1 mice at PN11 and PN17, but not at PN28 (Figure 5B).

### Vascular Endothelial and Hepatic Growth Factor Are Not Altered in the rd1 Model

**FIGURE 6:**
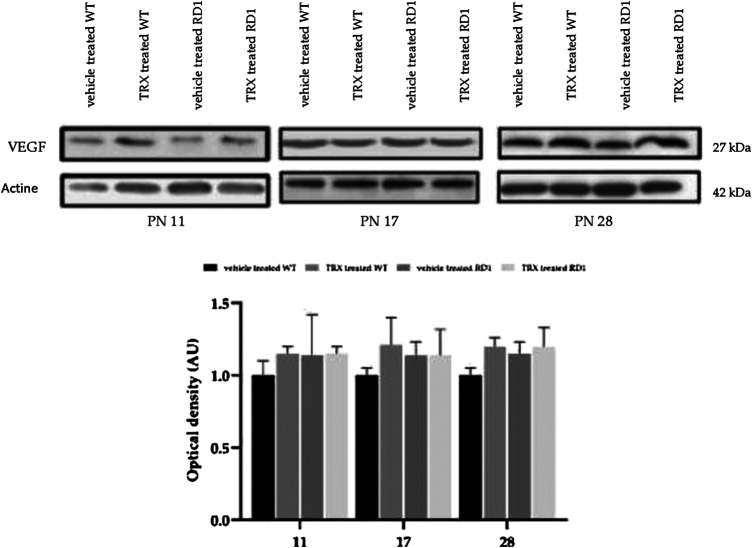
Retinal VEGF determination by western-blot. **(A)** Western-blot bands images for control and rd1 mice (n = 3). **(B)** Histogram represents the bands optical density quantification (VEGF/Actin ratio) in each group. The error bars represent the standard error of the mean. N = at least four in each experimental group.

VEGF is a trophic factor that increases endothelial cells survival, promotes proliferation and migration of endothelial cells and increases vascular permeability (Boehm et al., 1997). In addition, a decrease in VEGF has been reported in aqueous humor of RP patients (Salom et al., 2008). We have determined the retinal VEGF expression in WT and rd1 mice treated and non-treated with TRX, by western blot (Figures 6A,B). However, no differences were observed between VEGF expression in control and rd1 mice in none of the disease stages, moreover, no TRX effect was either observed.

**FIGURE 7:**
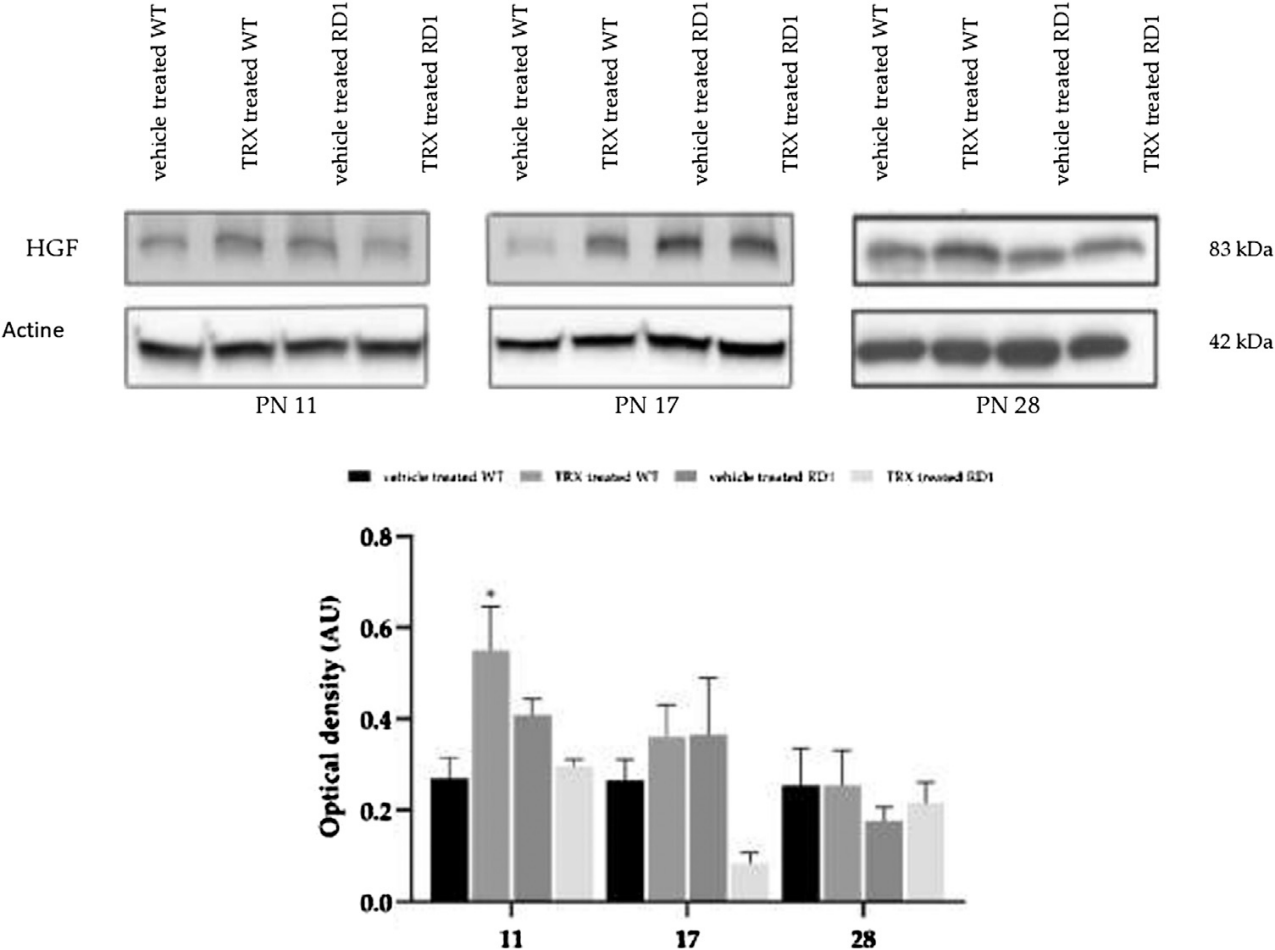
Retinal HGF determination by western blot. **(A)** Western-blot bands images for control and rd1 mice (n = 3). **(B)** Histogram represents the bands optical density quantification (HGF/Actin ratio) in each group. The error bars represent the standard error of the mean. (*p < 0.05 vs. all the other groups). N = at least four in each experimental group.

HGF is another factor that has been related with ocular angiogenesis. To date it has not been studied its role in RP. Our western blot results show that though there is a significant increase in HGF expression at PN11 in WT treated mice when compared with WT animals, no differences were observed in HGF between WT and rd1 mice (Figures 7A,B). Likewise, GFAP results, TRX treatment did not change HGF retinal expression in WT and rd1 mice at PN17 or PN28.

## Discussion

TRX is a well known antioxidant that has been shown to be effective in a wide variety of animal models with oxidative and inflammatory disorders (Nakamura et al., 2009). However, TRX effect in animal models of RP has not been studied to date. In this work, we have investigated if TRX was able to increase photoreceptor survival in the rd1 model of RP. Moreover, we also studied its effect to restore other retinal alterations which may be induced by photoreceptor death, such as changes in inflammatory and vascular markers.

### Thioredoxin Protects Photoreceptor Death in the rd1 Mice Retina

RP animal models, including the rd1 mouse model, present a rapid degeneration of photoreceptors that can be easily demonstrated with the help of the TUNEL assay. Several studies have described the way in which rods begin to degenerate in the rd1 mouse model and they all agree that rods degeneration peaks occurs around PN11-13 (Paquet-Durand et al., 2006) and that rods degeneration ends close to PN20 (Carter-Dawson et al., 1978).

The nuclei of photoreceptors are found in the ONL. The progressive deterioration of this layer of the retina, analyzed by measuring its thickness or by measuring the number photoreceptor rows in this layer, have been used in RP studies. However, though the maximum rate of cell death is produced around postnatal day 12 in this animal model, this cannot be demonstrated just determining the number of rows in the ONL. At PN11 and PN12 there is no difference in the ONL thickness in control and rd1 retina. In this sense, this parameter is not useful to determine the effect of different treatments at this post natal ages. For this reason we have decided to perform the TUNEL staining as it is a more precise technique to observe possible treatment effects.

Herein, we demonstrate that TRX treatment significantly decreases TUNEL positive photoreceptors in rd1 mouse retinas at PN11 in peripheral, medium and central retina (Figure 2). At PN17 TRX was only able to decrease the number of TUNEL positive cells in the rd1 far periphery retina (*p < 0.05) but could not decrease photoreceptor death in the medium and central retina (Figure 2B). This different response to TRX treatment of the different retinal areas studied may be related to the fact that there is a gradient in the rd1 degeneration, from the periphery to the central retina. Because of nearly no TUNEL staining could be observed in the retina of rd1 mice at PN28, probably due to the fact that at this stage rod degeneration is ended, we could not demonstrate any beneficial effect of TRX on cell survival at this age.

TRX protective effects have been recently reported in retinas exposed to perinatal hypoxia-ischemia (Holubiec et al., 2020) and in light-induced photoreceptor degeneration in diabetic mice (Kong et al., 2019). Some studies have also demonstrated the use of TRX and its effect to prevent cell death induced by cerebral ischemia (Hattori et al., 2004). Our results help us to conclude that TRX treatment may be helpful and slows down photoreceptor death in the first stages of the disease, while some rods are still present in the retina.

### Thioredoxin Effect on Glutathione Metabolism Alterations in rd1 Retina

Oxidative stress is related to the pathogenesis of many retinal diseases and it has also been suggested to be involved in the pathogenesis of RP, both in animal models and in patients (Shen et al., 2005). In this sense, the normalization of the redox state through the manipulation of endogenous levels of thiol antioxidants, for example, with TRX, could be an effective therapeutic strategy for RP.

The glutathione system is one of the antioxidant defense systems. Moreover, it is present in most mammalian cells and therefore it plays a key role in the eye (Riley et al., 1980). Under normal conditions, retinal GSH is almost exclusively confined to Müller cells, astrocytes and horizontal cells (Pow and Crook, 1995). Retinal GSH concentrations are maintained thanks to *de novo* synthesis, to the glutathione disulfide regeneration (GSSG) and to extracellular GSH capture (Circu and Aw, 2012).

Our results may also indicate a prevalent role of GSH at the beginning of the disease in rd1 mice. At PN11, GSH concentration was significantly decreased in rd1 retinas when compared with all the other groups (Figure 3A). Nevertheless, when retinal degeneration has progressed no differences could be observed between retinal GSH concentrations in WT and rd1 mice. TRX treatment increased the amount of GSH concentrations in the retina of all of rd1 mice. Results regarding GSSG may be confusing because we have observed increases in GSSG concentrations after TRX treatment (Figure 3B). New studies determining the activity of different GSH metabolism enzymes should be performed to further clarify the role of this increase in GSSG concentration. In this sense, it should be interesting to analyze glutathione peroxidase (GPx) retinal activity. This enzyme uses GSH as a cofactor to reduce hydrogen peroxide, resulting in the formation of GSSG. Still, our results confirm that TRX administration is beneficial, considering its effect on GSH metabolism as we have observed an increase in GSG/GSSG ratio in rd1 treated mice. This ratio is commonly used to measure the redox status of the cell, so that it reflects the cellular antioxidant capacity (Schafer and Buettner, 2001).

These results may suggest that, at least, part of the protective effect in the initial phases of the photoreceptor degeneration exerted by TRX, may be due in part to the GSH system activation. Indeed, there is considerable evidence showing that the maintenance of GSH cell stores protects against cell death (Hall, 1999). As reported in the introduction section TRX is and antioxidant protein that regulates thiol modifications and redox signaling. GSH plays an important role in modulating redox homoeostasis and its depletion is involved in multiple diseases. The administration of TRX to RP mice ameliorates retinal damage because it decreases oxidative injury and inhibits cell death.

### Thioredoxin Treatment Partially Reduces Retinal Macro and Microgliosis in rd1 Mice

Neuroinflammation is considered as a hallmark of numerous chronic degenerative disorders (Glass et al., 2010). The best-known aspect of the retinal glial response is that Müller cells upregulate GFAP, a protein that is considered as a marker for reactive gliosis, not only in the retina, but also in the CNS (Nakazawa et al., 2007). Müller cells are the predominant macroglial element of the retina, representing 90% of the glia. An increase in GFAP in glial cells has been demonstrated in both animal and human models of almost all retinal diseases, including glaucoma (Kim et al., 1998), retinal ischemia (Nishiyama et al., 2000) and in age-related macular degeneration (Guidry et al., 2002). Inflammation plays also a fundamental role in RP (Vecino et al., 2016). The glial reaction is thought to represent an attempt by the Müller cells to protect the retina from damage and to promote tissue repair. However, an acute activation of the macroglia has a neuroprotective effect, but continuous activation could be harmful.

As in the central nervous system (CNS), retinal microglial cells are sensors of possible disorders in the neuronal environment. Again, although microglial activation is commonly associated with neuroprotection, if activation is excessive or prolonged, it can lead to constant inflammation (Bignami and Dahl, 1979) and a chronic excessive activation, with serious pathological side effects. In RP, the interaction between neuronal damage and microglial activation generates a cycle that causes uncontrolled inflammation, contributing to the progression of disease (Gao and Hong, 2008).

To study the characteristics of Müller cells in the rd1 mouse retina we carried immunostainings of GFAP at different stages of retinal degeneration in RP. It has been shown that microglial cells express the ionized calcium binding adapter molecule 1 (Iba1). Therefore, Iba-1 staining was selected to study microglial activation.

Our results suggest that there is a reactive macro and microgliosis that is associated with degeneration in rd1 mice. We found statistically significant increases in GFAP and Iba1 in all areas of the retina, at all studied ages in vehicle treated rd1 mice (Figures 4,5). TRX treatment decreased GFAP reactivity in rd1 mice, but this effect was only observed at PN11. On the other hand, no TRX effect was observed on rd1 retinal macrogliosis at PN17 or PN28. Other authors have suggested that an increase in GSH may inhibit glial activation (Gouix et al., 2014), and this may explain the observed TRX effect on macroglia only at PN11.

Iba1 immunolabeling was upregulated in rd1 retinas compared to control retinas at all studied ages (Figures 5A,B). These results are similar to those found for GFAP. In the case of TRX, its effect was longer as it significantly reduced Iba1-positive immunolabelling in rd1 mice at PN11 and PN17.

Our results suggest that TRX may have protective effects at least in in the initial phases of the photoreceptor degeneration in this RP animal model. Oxidative stress is an important factor related to photoreceptor death in RP. Moreover, the manipulation of cellular redox status may be a therapeutic strategy to prevent also inflammation in RP. In this sense, TRX regulates oxidative stress because it increases the concentration of the antioxidant GSH and because of that it also combat inflammation. New studies should also be performed to be able to determine if increased doses of TRX may induce a longer beneficial effect.

### Vascular Endothelial and Hepatic Growth Factor Are Not Altered in the rd1 Model

VEGF is a growth factor that is involved in numerous retinal pathologies, such as proliferative diabetic retinopathy (Gupta et al., 2013), diabetic macular edema (Penn et al., 2008), etc. HGF plays an important role in ocular angiogenesis and in neuroprotection of retinal tissue. It has been shown that retinal ischemia and diabetic retinopathy increases the expression of HGF (Nishimura et al., 1999; Simó et al., 2006). Anyhow, we have not demonstrated any changes in the expression of these two factors in rd1 mice retina compared to control mice (Figures 6,7). For this reason, we have not been able to demonstrate any effect of TRX on the vascular changes that are usually observed in last phases of this retinal degeneration. We suggest that different vascular markers should be studied in order to demonstrate any therapeutically effects.

## Data Availability Statement

The raw data supporting the conclusions of this article will be made available by the authors, without undue reservation.

## Ethics Statement

The animal study was reviewed and approved by CEU Cardenal Herrera Universities Committee for Animal Experiments (reference 11/013).

## Author Contributions

Conceptualization, MM and IA; methodology, VH-R.; investigation, RG-H; AC; AF-C; writing—original draft preparation, MM; writing—review and editing, MM and TR. All authors have read and agreed to the published version of the manuscript.

## Funding

Cardenal Herrera CEU and University and San Pablo CEU Foundation University, grant numbers: INDI 19/35 and Consolidación 2018/19. Generalitat Valenciana (ACIF/2019/199) to A.C., Emergente (GV/2019/125) and Emergente (GV/2019/034) to VH-R.

## Conflict of Interest

The authors declare that the research was conducted in the absence of any commercial or financial relationships that could be construed as a potential conflict of interest.
